# Auxological, Clinical, and MRI Abnormalities in Pediatric Patients With Isolated Growth Hormone Deficiency

**DOI:** 10.7759/cureus.54904

**Published:** 2024-02-25

**Authors:** Naseem Y Alyahyawi

**Affiliations:** 1 Pediatric Department, Faculty of Medicine, King Abdulaziz University, Jeddah, SAU

**Keywords:** mri, neuroradiological assessment, clinical characteristics, auxological findings, children, growth hormone deficiency

## Abstract

Objective: This study aimed to assess the auxological, clinical, and MRI features of pediatric patients with isolated growth hormone deficiency (GHD) by analyzing the demographic and clinical characteristics of the study cohort.

Methods: A cohort of 115 pediatric patients diagnosed with isolated GHD was included. The patients were evaluated at a tertiary center in Jeddah, Saudi Arabia. Collected data included demographic information and auxological evaluations, such as height standard deviation (SD), height centile, weight SD, weight centile, and bone age SD. Neuroradiological assessments, particularly magnetic resonance imaging (MRI) of the hypothalamic-pituitary region, were collected to identify any structural abnormalities contributing to GHD.

Results: A total of 67 (SD 58.3) were males. The mean age was 9.55 years (SD 3.45). The mean bone age was 7.37 years (SD 3.24), indicating delayed bone development. Height measurements reflected a significant growth impairment, with a mean height SD of -2.45 (SD 1.12).

Out of the 115 pediatric patients in the study cohort, 84 (73%) underwent neuroradiological assessments using brain MRI. Among these, 12% were found to have MRI abnormalities. The prevalence of MRI abnormality in the subgroup with severe growth hormone deficiency was higher, reaching 21%. The peak growth hormone (GH) in the growth hormone stimulation test was 6.38 ng/mL (SD 3.24). There was a significant difference in the peak GH levels between the subgroup of patients with normal MRI findings (mean 6.02 ng/mL, SD 2.47) and those with abnormal MRI findings (mean 3.2 ng/mL, SD 2.8) (p=0.01).

Conclusion: Children with isolated GHD exhibited significant growth impairment and clinical characteristics consistent with the disorder. Neuroradiological abnormalities are common among patients with severe growth hormone deficiency; therefore, radiological assessment including MRI of the pituitary gland is recommended in patients with severe isolated growth hormone deficiency.

## Introduction

Growth hormone deficiency (GHD) in children is a complex endocrine disorder characterized by impaired linear growth, delayed skeletal maturation, and various physical and psychological complications [1,2]. It is crucial to thoroughly investigate the auxological, clinical, and neuroradiological features of children with GHD to ensure accurate diagnosis, appropriate management, and optimal outcomes [3,4].

Auxological evaluation plays a pivotal role in assessing growth impairment and potential underlying abnormalities in children with GHD. Various parameters are examined, including age, bone age, height standard deviation (SD), height centile, weight SD, weight centile, mid-parental height, and insulin-like growth factor 1 (IGF1) levels [3,5]. GHD can present at any age during childhood [6]. Bone age assessment is particularly valuable in determining the degree of skeletal maturation delay, which is a common feature in children with GHD [7]. Height SD and centile provide valuable insights into the severity of growth impairment and can help guide treatment decisions [8]. Evaluation of weight SD and centile aids in monitoring overall body composition and identifying potential concurrent nutritional issues [9]. Mid-parental height, often utilized as a reference, helps assess the genetic potential for height [10]. Lastly, measurement of IGF1 levels provides important information regarding growth hormone secretion and has been shown to correlate with growth outcomes in children with GHD [2].

Beyond auxological features, a comprehensive clinical approach is essential in diagnosing and managing GHD in children. This includes meticulous history-taking to identify underlying causes, such as genetic disorders, chronic illnesses, radiation therapy, or traumatic brain injury [11]. Comprehensive physical examinations are performed to identify physical stigmata that may be indicative of specific genetic syndromes, coexisting endocrine abnormalities, or associated dysmorphic features [12]. Furthermore, hormonal evaluations may be conducted to assess the function of other pituitary hormones, such as thyroid-stimulating hormone, adrenocorticotropic hormone, and gonadotropins, and deficiencies in these hormones may coexist with GHD [13].

In addition to auxological and clinical evaluations, neuroradiological investigations are crucial in children with GHD. Magnetic resonance imaging (MRI) of the hypothalamic-pituitary region allows for the assessment of the pituitary gland, stalk, and surrounding structures, aiding in the identification of structural abnormalities that can contribute to GHD [14]. Common radiological abnormalities observed include the partial or complete absence of the pituitary gland, pituitary stalk interruption syndrome, septo-optic dysplasia, and craniopharyngiomas [15]. Abnormalities detected by neuroradiological evaluation can help guide further therapeutic decisions and provide insights into the underlying pathogenesis leading to GHD [16].

## Materials and methods

A retrospective analysis of 115 children diagnosed with isolated GHD between January 2008 and March 2021 was conducted. All participants met the diagnostic criteria for isolated GHD, including short stature (height {HT} less than 2 standard deviation score {SDS}) and inadequate growth velocity, as determined by pediatric endocrinologists. The GH stimulation test was done to confirm the diagnosis of GHD using any two of the following GH stimulants: glucagon, clonidine, and insulin. The GH stimulation test followed a standardized testing protocol. In the case of insulin stimulation, a dose of 0.1 IU/kg of insulin was administered, and GH samples were drawn at intervals of 0 min, 15 min, 30 min, 45 min, 60 min, 90 min, and 120 min. For glucagon stimulation, a dose of 0.03 mg/kg (up to a maximum of 1 mg) was used, and GH samples were drawn every 30 min for up to 180 min. In the case of clonidine stimulation, a dose of 0.15 mg/m^2^ (up to a maximum of 0.25 mg/m^2^) was used, and GH samples were drawn every 30 min for up to 120 min. Isolated GHD was defined as a peak GH level <10 ng/mL in the absence of other pituitary hormone deficiencies. Severe growth hormone deficiency was defined as a peak GH level <3 ng/mL.

Demographic and clinical characteristics, including age, gender, bone age, height standard deviation (SD), height centile, weight SD, weight centile, mid-parental height, IGF1 levels, peak growth hormone deficiency, and bone age SD, were assessed. Neuroradiological findings were also reviewed. Other hormones that were routinely tested included thyroid stimulating hormone (TSH), and free thyroxine (FT4) was done in all patients. Morning cortisol, luteinizing hormone (LH), and follicle-stimulating hormone (FSH) levels were measured if it was clinically indicated.

The data were analyzed using SPSS version 21 (Armonk, NY: IBM Corp.). For categorical data, mean and standard deviation were calculated, while numerical data were presented as numbers and percentages. A p-value of <0.05 is considered significant.

## Results

The demographic and clinical characteristics of the study subjects are presented in (Table 1). The majority of children were aged ≤10 years and 67 (58.35%) were male. The mean age was 9.55 years (range: 3-17 years), with a mean bone age of 7.37 (SD 3.24) years. The gap between chronological age and bone age was 1.69 years (SD 1.56), indicating delayed bone development. The mean height SD was -2.45 (SD 1.12), indicating significant growth impairment. The mean peak growth hormone was 6.38 (SD 3.24) ng/mL.

**Table 1:** Descriptive statistics of demographic and clinical characteristics of 115 study subjects with isolated GHD. IGF: insulin-like growth factor; FT4: free thyroxine; GHD: growth hormone deficiency

Characteristics	No. (%)	Mean (SD)
Age groups (<10 years)	67 (58.3)	-
Age groups (>10 years)	48 (41.7)	-
Male	67 (58.3)	-
Female	48 (41.7)	-
Age	-	9.55 (3.45)
Bone age	-	7.37 (3.24)
Delay between chronological age and bone age	-	1.69 (1.56)
Height (SD)	-	-2.45 (1.12)
Height (cm)	-	122.27 (16.84)
Weight (SD)	-	-2.03 (1.76)
Weight (kg)	-	25.74 (11.42)
Mid parental height (cm)	-	162.64 (8.01)
IGF1	-	142.23 (108.79)
Peak growth hormone (ng/mL)	-	6.38 (3.24)
FT4	-	1.38 (0.77)
Bone age (SD)	-	-2.05 (1.43)
IGF Z score (SD)	-	-1.25 (1.60)

Neuroradiological assessments were conducted on 87 pediatric patients with confirmed isolated growth hormone deficiency (GHD). Among these patients, nine out of 87 (12%) were found to have neurohypophyseal abnormalities. Additionally, in a subgroup of 28 pediatric patients with severe growth hormone deficiency (defined by a peak growth hormone level of <3 ng/dL in the growth hormone stimulation test), it was found that six out of 28 patients (21%) in this subgroup had neurohypophyseal abnormalities, indicating a higher prevalence compared to the overall pediatric patient population with isolated GHD (Table 2). A detailed description of the neurohypophyseal abnormalities is summarized in (Table 3).

**Table 2:** Prevalence of neurohypophyseal abnormalities in 28 pediatric patients with severe GHD who underwent MRI brain. The data has been represented as n (%). GHD: growth hormone deficiency

MRI result	No. of patients with severe GDH (%)
Normal MRI	22 (78.6%)
Neurohypophyseal abnormalities in MRI	6 (21.4%)

**Table 3:** Neurohypophyseal abnormalities detected by MRI in nine patients with GHD. GHD: growth hormone deficiency

Total number of patients with GHD and neurohypophyseal abnormalities on MRI	9
Hypoplastic anterior pituitary gland	4
Ectopic posterior pituitary	1
Rathe's cleft cyst	3
Adenohypophyseal microadenoma	1

A subgroup of patients who underwent MRI assessment to assess the severity of GHD was analyzed. Our analysis revealed that the mean peak GH level in patients with isolated GHD and abnormal MRI findings was 3.2 ng/mL (SD 2.8), which was statistically significantly lower than peak GH in patients with isolated GHD and normal MRI findings, which was 6.02 ng/mL (SD 2.47) (p=0.0128) (Table 4).

**Table 4:** Result of growth hormone stimulation test in the 87 pediatric patients with GHD who underwent MRI brain. P-value <0.05 is considered significant. GHD: growth hormone deficiency

MRI findings	No. of patients with GHD	Peak GH, mean (±SD)	p-Value
Normal MRI	78	6.02 ng/mL (2.47)	0.0128
Neurohypophyseal abnormalities in MRI	9	3.2 ng/mL (2.8)	

## Discussion

The findings of this study confirm that children diagnosed with isolated GHD in our cohort had significant growth impairment, as reflected by the mean height SD of -2.45 (1.12) [2]. This highlights the importance of early identification and appropriate management of isolated GHD to optimize growth outcomes.

Delayed bone age, as seen in this study, is consistent with previous findings and is a well-known feature in children with isolated GHD. Assessment of bone age is critical in determining the extent of growth retardation and helps in monitoring the response to growth hormone therapy [7].

Various types of neurohypophyseal abnormalities have been identified in MRI scans of patients with isolated GHD. Similar to our findings, the most common type of abnormality reported in the literature in patients with isolated GHD is pituitary hypoplasia, which is characterized by a small or underdeveloped pituitary gland [17]. In a study reported by Chen et al., pituitary hypoplasia was the most frequent MRI abnormality in their study on pediatric patients with GHD [18,19]. Other less common abnormalities reported include pituitary stalk interruption syndrome (PSIS), ectopic posterior pituitary, and absence or hypoplasia of the pituitary gland, as reported by Marziali et al. [12].

Neuroradiological assessments in a subset of cases with severe isolated GHD in our cohort revealed a higher prevalence of structural abnormalities in the hypothalamic-pituitary region. This finding is consistent with previous studies. In a study by Jagtap et al., it was found that patients with neuroradiological abnormalities had more severe characteristics of GHD [16]. These findings support the role of neuroradiology in identifying underlying anatomical or structural causes of GHD, prompting appropriate therapeutic interventions [19,20].

The present study has certain limitations that should be acknowledged. First, as a retrospective study, we relied on existing medical records for data collection. Additionally, the study was conducted at a single center, which could introduce bias and limit the generalizability of our findings to a larger population. The lack of data on consanguinity and the lack of genetic analysis are other limitations of our study. Also, the sample size in our study was modest, potentially limiting the statistical power and precision of our results. Finally, we focused this study on children with isolated GHD and did not include data on other pituitary hormone deficiencies. It is important to note; however, that a percentage of children with isolated growth hormone deficiency may develop multiple pituitary hormone deficiencies in the future. Therefore, after establishing the diagnosis of isolated growth hormone deficiency, additional testing of the pituitary hormonal axis should be conducted.

## Conclusions

The present study adds to the growing body of evidence demonstrating the higher prevalence of pituitary abnormalities among patients with isolated severe GHD, aligning with previous studies and indicating the pivotal role of neuroimaging in the evaluation of these individuals.

The collective evidence emphasizes the necessity of MRI evaluation to identify and characterize neurohypophyseal abnormalities in patients with isolated severe GHD. These structural abnormalities have important implications for the underlying etiology and management approach. Therefore, the integration of neuroradiological assessments, particularly MRI of the pituitary gland, is crucial in the comprehensive evaluation of patients with severe growth hormone deficiency to identify any structural anomalies that may contribute to the condition. Future research should focus on further understanding the underlying mechanisms and exploring novel treatment modalities to improve the overall care and outcomes for patients with isolated GHD.
